# Trajectories of Immediate and Delayed Verbal Memory in the Spanish General Population of Middle-aged and Older Adults

**DOI:** 10.3390/brainsci10040249

**Published:** 2020-04-22

**Authors:** Ivet Bayes-Marin, Daniel Fernández, Elvira Lara, Natalia Martín-María, Marta Miret, Darío Moreno-Agostino, José Luis Ayuso-Mateos, Albert Sanchez-Niubo, Josep Maria Haro, Beatriz Olaya

**Affiliations:** 1 Research, Innovation and Teaching Unit, Parc Sanitari Sant Joan de Déu, Sant Boi de Llobregat, 08830 Barcelona, Spain; i.bayes@pssjd.org (I.B.-M.); albert.sanchez@pssjd.org (A.S.-N.); jmharo@pssjd.org (J.M.H.); beatriz.olaya@pssjd.org (B.O.); 2 Centro de Investigación Biomédica en Red de Salud Mental (CIBERSAM), Instituto de Salud Carlos III, 28029 Madrid, Spain; natalia.martinm@uam.es (N.M.-M.); marta.miret@uam.es (M.M.); dario.moreno@uam.es (D.M.-A.); joseluis.ayuso@uam.es (J.L.A.-M.); 3 Department of Medicine, Universitat de Barcelona, 08036 Barcelona, Spain; 4 Serra Húnter fellow, Department of Statistics and Operations Research, Polytechnic University of Catalonia-BarcelonaTech, 08028 Barcelona, Spain; 5 Department of Psychiatry, Hospital Universitario de La Princesa, Instituto de Investigación Sanitaria Princesa (IIS-Princesa), 28006 Madrid, Spain; elvira.lara@uam.es; 6 Department of Psychiatry, Universidad Autónoma de Madrid, 28029 Madrid, Spain

**Keywords:** cognition, cognitive functions, cognitive aging, memory, epidemiology

## Abstract

(1) Cognitive decline differs among individuals and cognition function domains. We sought to identify distinct groups of immediate and delayed verbal memory in two age subsamples (50–64, 65+ years), and to analyze associated factors. (2) Latent class mixed models were used to identify verbal memory trajectories in a sample of Spanish community-dwelling individuals over 8 years’ follow up. Chi-square and Kruskal–Wallis tests were used to assess differences among trajectories. (3) Different trajectories were identified. In the case of immediate verbal memory, these were: very low/decline (6.3%), low/stable (38.2%), medium/slow decline (43.4%), and high/slow decline (12.2%) in the middle-aged group, and low/decline (20.4%), medium/slow decline (60.4%), and high/slow decline (19.2%) in the older subsample. In delayed verbal memory, more distinct patterns were found: very low/decline (12.4%), low/stable (51.4%), medium/accelerated decline (24.7%), and high/slow increase (11.4%) in the younger group, and low/slow decline (34.4%), medium/decline (52.7%), and high/slow decline (12.9%) in the older group. (4) Overall, low initial performance and decline were associated with older age, lower education, and higher diabetes/stroke prevalence. Differences found suggests heterogeneity in cognitive ageing. The high prevalence of cardiovascular diseases in those with worse cognition suggests that early interventions to prevent those conditions should be targeted in midlife to delay cognitive decline.

## 1. Introduction

By 2050, the world’s population aged 60 years and older is expected to almost double, from 12.7% in 2017 to 21.3% [1]. Europe is projected to remain the most aged region, with 34% of the population aged 60+ [1]. Along with this rapid increase, [2], there is huge burden placed on older individuals, their families, and society in general [3]. Cognitive function, however, is expected to decline along with normal aging [4], but the rate by which cognition declines seems to be distinct across individuals [5]. Understanding these age-related changes in cognition and their determinants is important because an optimal cognitive function in older adults can help maintain functional their independence and effective communication with others [6,7]. 

Longitudinal studies have shown that around the age of 60, cognition is more likely to begin to decline [8], although there seems to exist important heterogeneity in the rate of decline. The use of latent class analysis techniques to classify older adults according to their trajectories of cognition over time has overall revealed the presence of at least three [9,10], if not four [11,12,13] groups. Despite the fact that the differences between studies might be explained by distinct methodologies (i.e., sampling methods, outcomes, covariates), some commonalities can be found. First, the majority of the individuals maintain a stable trajectory or a slow decline, whereas a small part of them could be classified as rapid decliners [9,11,12]. Second, those with low baseline cognition scores are more likely to decline faster [10,11,12]. Third, those groups with better initial performance on cognition are composed mainly of younger individuals with higher education levels [10,11,12]. 

There is a suggestion that different cognition domains decline differently [13,14]. Crystallized intelligence, composed of verbal abilities, general knowledge, and number skills, is more likely to remain or gradually improve with age, whereas fluid intelligence, made up of other cognitive abilities such as memory, executive function, and processing speed, is more prone to decline with age [13,14,15]. Previous studies on verbal memory trajectories [11,12] have typically combined immediate and delayed verbal memory measures into one dimension, in spite of there being measures of different cognitive functions, short-term memory and long-term memory, respectively, according to the Shiffrin and Atkinson model of human memory [16]. 

Furthermore, several predictors of cognitive decline have been studied, and some factors have consistently been associated with faster decline of cognition. Medical conditions such as diabetes, cardiovascular disease, chronic obstructive pulmonary disease (COPD), and stroke have been shown to be associated with poorer cognitive performance [3,17]. Similarly, depression has been widely recognized as a risk factor for cognitive impairment [3,13]. Smoking status or ever having smoked have been associated with greater cognitive decline, as has physical inactivity, suggesting that active individuals have better cognitive function than sedentary ones [18,19,20]. Nonetheless, some sociodemographic variables, like sex, socioeconomic status, and level of education, have yielded inconsistent results [13,21]. 

Understanding cognitive decline related to age in middle-aged and older individuals and the factors involved in that decline are crucial for early interventions, particularly in earlier ages when preventive programs could be more effective in reversing or mitigating cognitive deterioration in order to delay care dependency. The aims of the present study were to: (1) identify different verbal memory trajectories in a community-dwelling sample of middle-aged (50–64) and older adults (65+), (2) determine the associations between several variables, such as sociodemographics, lifestyles, depression, and medical conditions in these trajectories.

## 2. Materials and Methods

### 2.1. Study Design and Data Collection

We analyzed data from “Edad con Salud”, a longitudinal survey of the Spanish general adult population (aged 18+ years with oversampling of those aged 50+ years). The first wave was part of the Collaborative Research on Ageing in Europe (COURAGE in Europe) study [22]. The baseline sample was nationally representative since it was generated by multistage clustered sampling and strata included all autonomous communities in Spain except Ceuta and Melilla. Data on households were provided by the Spanish Statistical Office. This study was conducted in accordance with the Declaration of Helsinki, and the study protocols for the baseline, wave one and two were approved by the Ethics Review Committee of Hospital Universitario La Princesa, Madrid (reference numbers PI-364, PI-2399, and PI-2801), and the Ethics Review Committee of Fundació Sant Joan de Déu (reference numbers PIC-12-11 and PIC-129-17). Informed consent was obtained from all individuals. Face-to-face structured interviews were conducted by trained lay interviewers at the respondents’ homes. 

Respondents unable to undertake the interview because of severe cognitive (MMSE score < 16 [23] or family’s report of dementia diagnosis) or physical impairment were administered a shorter version of the questionnaire to a proxy respondent.

**Figure 1:**
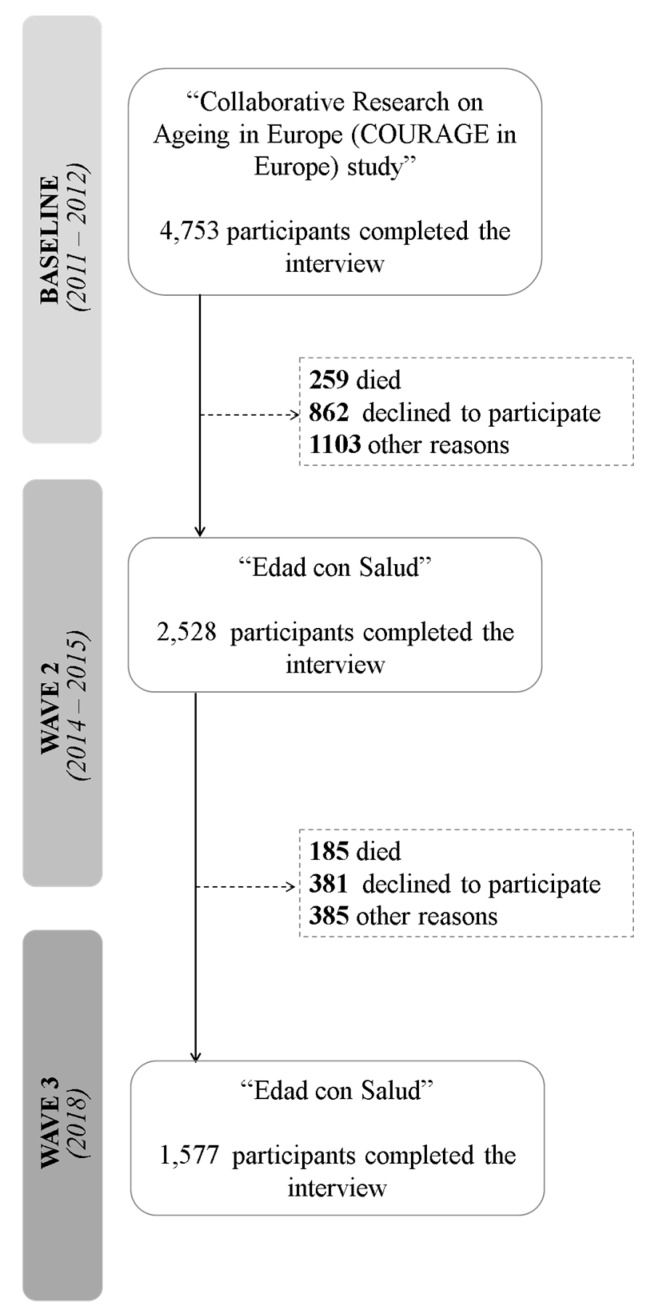
Flow-chart of the study population.

A total of 4753 persons participated at baseline between 2011 and 2012, with a response rate (RR) of 69.9%. The second wave (RR = 69.5%) took place between 2014 and 2015 and 2528 participants (53.2%) completed the interview, whereas in the third assessment (RR = 73.0%), which was undertaken in 2018, 1577 participants completed the interview. Figure 1 shows the study flowchart.

We used data from individuals who were aged 50 years or older at baseline. We included those participants who took part in the three assessments and completed a non-proxy interview (*n* = 1237), whereas those who had one or more missing values in immediate and/or delayed verbal recall in any wave were excluded (*n* = 148), resulting in a final analytical sample of 1089 individuals. 

### 2.2. Measures

To evaluate immediate and delayed verbal recall in the three waves, participants were asked to recall a list of 10 words four times immediately and after a short delay, respectively (Consortium to Establish a Registry for Alzheimer’s Disease (CERAD) [24]). A composite score was built as the sum of the number of correct words (0–30) for immediate verbal memory. Delayed recall scores ranged from 0 to 10. In both cases, higher scores indicated better memory. 

Respondents were asked about the presence of the following chronic conditions (CCs) in each wave: diabetes, hypertension, asthma, COPD, arthritis, angina pectoris, stroke, and depression. To assess these conditions, we used a combined method consisting of self-reported diagnosis in the previous 12-months, and/or symptom-based algorithms based on the World Health Organization Study on global AGEing and adult health WHO-SAGE protocol [25]. Depression was assessed with an adapted version of the World Health Organization Composite International Diagnostic Interview (CIDI) [26]. Presence or absence of these conditions was coded as *yes/no*. The number of conditions was arrived at by adding up the occurrences of all the above-mentioned CCs at baseline. 

Self-reported sociodemographic variables included sex, age, marital status (never married, married or currently cohabiting, separated or divorced, and widowed), level of education (less than primary education, primary education, secondary education, and tertiary education), quintiles of household income (first quintile indicating lowest level), ever worked coded as *yes/no*, and living in a rural or an urban area. 

Physical activity level (low, moderate, or high) was based on the Global Physical Activity Questionnaire version 2 (GPAQ v2) [27]. A 4-level tobacco consumption variable (never smoked, daily smoker, not daily smoker, and not current smoker) and a 4-category alcohol intake variable (lifetime abstainer, occasional drinker, infrequent heavy drinker, and frequent heavy drinker) were also included as covariates. 

The level of disability was assessed using the 12-item interviewer-administered version of the World Health Organization Disability Assessment Schedule 2.0 (WHODAS 2.0) [28]. Participants were asked to report the level of difficulty they had in doing various activities during the previous 30 days using a five-point scale. The global score was obtained as the sum of the items and transformed into a 0 to 100 scale, with higher scores indicating greater disability. 

Quality of life was measured with the WHOQOL-AGE, a version of the World Health Organization Quality of Life instrument (WHOQOL) that was developed for the old-age population [29]. 

### 2.3. Statistical Analyses

All analyses were calculated separately for people aged 50–64 (*n* = 633) and those aged 65+ (*n* = 456) years old at baseline, to explore differences between the two age groups and allow comparability with previous studies on verbal memory trajectories. Models were run independently for immediate and delayed verbal memory. 

Latent class growth analysis (LCGA), or also called the group-based trajectory model (GBTM) [30], were conducted on each age group using the Mplus version 7.4 software [31]. This method classifies individuals into groupings with similar patterns, called latent classes. The time metric was years of the study (0,3,6), and the outcome was immediate and delayed verbal memory assessed in waves 1–3. 

The number of trajectories was determined by analyzing 2–5 group models without covariates. Model selection to determine the optimal number of latent trajectories was performed according to the adjusted Bayesian information criterion (aBIC), where the lowest value indicates better fit [32]. Average posterior probabilities above 70% also indicate optimal fit [33]. Entropy index values higher than 0.80 indicate that the latent trajectories are highly discriminating [32]. Moreover, class sample sizes were also taken into account since inadequate sample size can lead to convergence problems, insufficient power to identify classes, and changing solutions [33]. Individuals were allocated to clusters according to maximum a posteriori criterium (MAP). 

The Shapiro–Wilk test was used to assess the normality assumption and Levene’s test to assess the equality of variances for a variable calculated for two or more groups. Categorical variables were compared using the Chi-square test or Fisher’s exact test, and continuous variables were compared using Kruskal–Wallis test to assess differences between latent classes. A two-sided *p*-value < 0.05 was considered statistically significant. These analyses and the graph display tasks were carried out using R Version 3.5.3 [34]. 

## 3. Results

### 3.1. Descriptive Analysis

Table 1 summarizes the baseline characteristics of each group subsample: 50–64 and 65+. The average baseline age was 56.62 [IQR = 60.00, 53.00] in the middle-aged group and 73.01 [IQR = 77.00, 68.00] in the older group; the sample comprised slightly more women than men (52.0% and 54.6%, respectively), and in the 65+ group, a higher proportion of participants had less than primary education (46.5%). The most prevalent conditions were hypertension (34.3% and 49.1%) and arthritis (19.6% and 33.4%, respectively). Disability was higher in the older group (15.02 [IQR = 25.00, 0.00]) compared to the younger (7.60 [IQR = 8.33, 0.00]). Middle-aged individuals showed higher scores on both measures of verbal memory (17.36 [IQR = 21.00, 14.00]) and 5.35 [IQR = 7.00, 4.00], respectively) in contrast with the 65+ subsample (13.41 [IQR = 16.00, 10.00] and 3.74 [IQR = 5.00, 2.00], respectively).

### 3.2. Verbal Memory Trajectories 

Table 2 shows the aBIC and entropy values for immediate and delayed verbal memory with two to five latent trajectory groups for both age subsamples. In the immediate verbal memory models, the four-group solution was selected as the best-fit model in the younger subsample (50–64), whereas in the 65+ subsample, the three-group solution was chosen. Similarly, in delayed verbal memory, the four-group solution was selected for the 50–64 subsample and the three-group solution for the 65+ subsample. Posterior probabilities were all above 0.70, indicating good fit.

**Figure 2:**
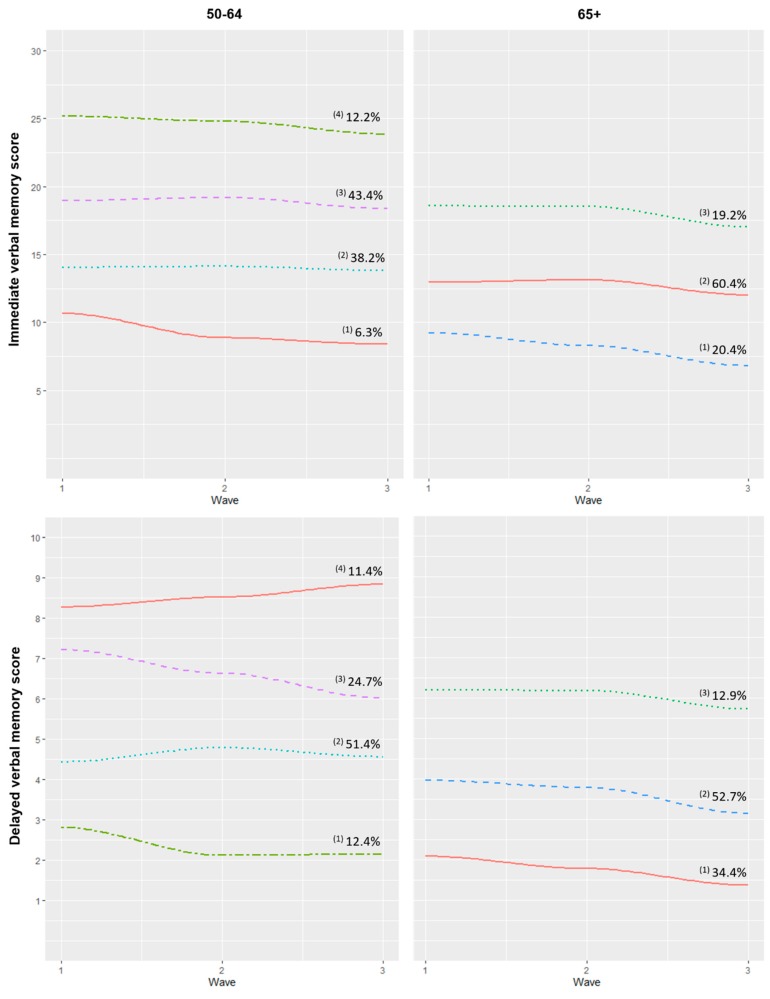
Trajectories of immediate and delayed verbal memory according to age. Note. The immediate verbal memory score ranges from 0 to 30, and delayed verbal memory ranges from 0 to 10, with higher scores indicating better performance. These trajectories were calculated using ‘time’ as a time-varying covariate. Immediate verbal memory 50–64: (1) Very low/decline, (2) Low/stable, (3) Medium/slow decline, (4) High/slow decline; 65+: (1) Low/decline, (2) Medium/slow decline, (3) High/slow decline. Delayed verbal memory 50–64: (1) Very low/decline, (2) Low/stable, (3) Medium/accelerated decline, (4) High/slow increase; 65+: (1) Low/slow decline, (2) Medium/decline, (3) High/slow decline.

Trajectory groups were identified and labelled according to their baseline status and decline in verbal memory over the three waves (Figure 2). Note that these labels were merely descriptive and did not imply clinical meaning. In the case of immediate verbal memory and the 50–64 subsample, a small proportion (6.3%) was classified into the “very low/decline” group, which showed very low scores at baseline and an evident decline rate. The second group (“low/stable”) was composed of 38.2% of the sample and presented low scores which remained stable over the waves. The third (“medium/slow decline”) and fourth (“high/slow decline”) groups presented medium and good scores and a slow decline pattern, with a proportion of 43.4% and 12.2% of the individuals in each group, respectively. In spite of the number of latent trajectories and their proportions, analogous groups were found in the 65+ subsample, where 20.4% were classified into the “low/decline” group, 60.4% into the “medium/slow decline” pattern, and 19.2% into the “high/slow decline” trajectory. 

Concerning delayed verbal memory trajectories, four patterns were identified in the middle-aged subsample. A small proportion of participants (12.4%) were classified into the “very low/decline” trajectory. Most of the sample (51.4%) was in the “low/stable” group, composed of individuals with low scores at baseline with a slight increase in the second wave which reverted in the third assessment. The greatest decline was found in the “medium/accelerated decline” pattern, composed of 24.7% of the sample. Finally, 11.4% of the participants, classified into the “high/slow increase” group, showed a slight rise in delayed verbal memory scores over the waves. In the case of the 65+ subsample, three trajectories of decliners with different initial performance were found: “low/slow decline” (34.4%), “medium/decline” (52.7%), and “high/slow decline” (12.9%).

### 3.3. Association between Verbal Memory Trajectories and Covariates

Table 3 and Table 4 present the results of comparisons of demographic and clinical characteristics at baseline in group membership for immediate verbal memory. Similar results were found for both age subsamples. Those classified into “very low/decline” patterns showed higher age (50–64: 58.41 [IQR = 61.5, 55.5], *p* < 0.001; 65+: 76.10 [IQR = 79.3, 73.0], *p* < 0.001) and greater proportion of participants with less than primary education (48.4%, *p* < 0.001; 67.8%, *p* < 0.001). In contrast, those participants classified into both “high/slow decline” profiles had higher proportions of individuals with tertiary education (53.4%, *p* < 0.001; 19.5%, *p* < 0.001, respectively), people who had worked (100.0%, *p* = 0.001; 92.7%, *p* = 0.001), and those with a higher household income (36.9%, *p* < 0.001; 20.8%, *p* < 0.001).

Regarding their clinical profiles, the middle-aged “very low/decline” group had a greater number of diseases (1.52 (SD = 1.23), *p* < 0.001) compared to the other profiles. Most notably, prevalence of diabetes (22.6%, *p* < 0.001), arthritis (29.0%, *p* = 0.001), stroke (9.7%, *p* = 0.037), and depression (25.8%, *p* = 0.002) were significantly higher in this group. In the case of the same profile in the 65+ subsample, only diabetes (32.2%, *p* = 0.007) and arthritis (44.8%, *p* = 0.031) prevalence reached statistical significance, both being higher in the “very low/decline” pattern. These profiles also showed greater disability (12.72 (SD = 19.12), *p* < 0.001; 24.10 (SD = 22.27), *p* < 0.001) and worse quality of life (63.34 (SD = 17.34), *p* < 0.001; 66.15 (SD = 13.66), *p* < 0.001) in both age groups. 

Concerning lifestyles, those classified into “very low/decline” groups had greater proportions of low physical activity level (48.4%, *p* = 0.001; 41.5%, *p* = 0.004), whereas those who had better performance on cognition at baseline in both age groups showed higher levels of physical activity (53.4%, *p* = 0.001; 39.0%, *p* = 0.004). Tobacco and alcohol were significant only in the 65+ subsample. The participants classified into “very low/decline” had a higher proportion of ‘never smoked’ participants (73.6%, *p* = 0.013) as well as lifetime abstainers (23.2%, *p* = 0.023). 

In the same way, Table 5 and Table 6 display the results for delayed verbal memory. Comparable trends and associations were found regarding age, level of education, household income, urbanity, disability, quality of life, and physical activity. Nevertheless, some differences should be highlighted. The pattern “high/slow increase” had a higher proportion of women (71.0%, *p* < 0.001) compared to the other trajectory groups in the middle-aged subsample. In delayed verbal memory, marital status was significant (*p* = 0.013) in the older subsample, where participants classified into “low/slow decline” group had a widowed participants proportion of 40.6%. Only those participants classified into the “very low/decline” pattern showed a higher prevalence of diabetes (20.6%, *p* = 0.037) and stroke (8.8%, *p* = 0.010) compared to the other trajectories. 

## 4. Discussion

Our aim was to determine the number of distinct latent groups in a population-based sample of middle-aged and older adults using two cognitive outcomes (i.e., immediate and delayed recall) and identify whether these groups differed according to several variables. 

Four and three distinct trajectories of immediate recall were identified in the middle-aged and the older subsample, respectively. Even though not great differences have been found in terms of rates of decline, our findings suggest that having low scores at baseline is followed by a greater rate of decline over time, whereas having medium and higher scores at baseline is related to a slower decline afterward. These results are in keeping with previous studies, where poor initial performance was associated with greater decline [10,11,12].

As for the delayed recall test, we found that 11.4% of the middle-aged participants followed a “high/slow increase” trajectory, with higher scores at baseline and a slight increase over time. This improvement might be the result of the re-test or practice effect, which is especially common in fluid intelligence measures such as verbal episodic memory [35]. This effect is generally more pronounced in the initial assessments and is expected to diminish over subsequent waves [36]. This effect was also observed in the “low/stable” group, with a subtle improvement in wave two that reverted in the third assessment. Moreover, some authors suggest that the retest effect varies across different cognitive tests and cognitive domains [36]. This might explain why this trend was not observed when using immediate recall as the outcome. 

Comparison between latent groups suggests different patterns. In general, those individuals classified in “very low/decline” patterns are more likely to be older than those with better baseline scores. Likewise, the participants with better cognition included a higher proportion of people with tertiary education. Thus, education level seems to contribute to the initial levels of cognition. Higher education might contribute to greater cognitive reserve to compensate brain neuropathology and delay the onset of clinical symptoms [37]. Similarly, better household income might be related to more cognitively demanding occupations, and better access to health services, also more likely when living in an urban area, with a positive effect on cognition [38]. 

Our findings also show that cardiovascular conditions such as diabetes and stroke are associated with lower memory scores in the 50–64 age group for both verbal memory measures. The individuals classified in the “very low/decline” and “low/stable” trajectories for both verbal memory measures showed a remarkably higher prevalence of those conditions, which are causally related to neuronal loss and which are associated with cognitive decline [3,39]. However, only diabetes remained significantly associated with immediate memory in the older subsample, whereas none of the studied CCs was associated with delayed verbal memory. This is in line with previous studies showing that cardiovascular risk factors and conditions are related to cognitive decline in midlife [40,41]. 

Presence of arthritis was also associated with declining trajectories of immediate verbal memory for both age groups. Some authors suggest that cardiovascular diseases increase the risk of arthritis, and acting synergistically may accelerate cognitive deterioration [42,43]. In our data, depression was associated only with immediate verbal memory in the middle-aged sample. One explanation for why it has not been found in delayed verbal memory is the effect of affected attentional processes which are common in depressed individuals and which play an important role in immediate or short-term memory [16]. Moreover, memory appears not to be affected by depression among the oldest cohort, suggesting that at this age, other underlying factors influence cognition. 

As expected, those with lower verbal memory scores showed greater disability, and consequently reported a poorer quality of life. These results were common between the two age subsamples. Regarding the health-related risk factors, of note is the association between physical activity and cognition trajectories. Those who scored higher in cognition (high and medium performers) engaged more frequently in vigorous physical activity, whereas those with very low scores of cognition in the first wave reported lower physical activity levels. This is in keeping with emerging research focused on the role of physical activity in cognitive function, suggesting that active individuals have better cognitive function than sedentary ones [18,19,20]. Moreover, there is some evidence in the literature about protective effects of physical activity on risk for future dementia [44]. Conversely, smoking status and alcohol consumption yielded paradoxical and mixed evidence. The literature suggests that current smokers are more likely to present cognitive decline, though in our 65+ sample, they were the ones who had better immediate verbal memory scores and a slower decline pattern [45]. In the same line, the same participants showed a greater proportion of heavy and occasional drinkers. One possible explanation is the effect of their health status on lifestyles, so those with a higher prevalence of CC would quit smoking or reduce alcohol intake to improve or at least not worsen their health. 

### Strengths and Limitations

Our findings should be considered in light of limitations. First, the presence of CCs was partially based on self-reporting, although we also used symptom-based algorithms to avoid underdiagnosis. Nevertheless, some authors support self-reported diagnostics as a well-established method to evaluate CC in population-based studies [46]. Second, we analyzed data from those individuals who participated in the three waves, and who had complete information for verbal memory variables. This might have introduced some bias since participants with worse health and cognition status may have been likelier to drop out of the study or to have missing values in the cognitive tests [47]. Related to this, participants who completed a proxy interview due to cognitive problems, such as neurodegenerative disease, were excluded from the analyses, since we aimed to study non-clinical cognitive decline. Nonetheless, participants with the incipient neurodegenerative disease may have been included in our analytical sample because of the sensitivity of the used screening tool. Third, it would have been of interest to assess the effect of practice on the estimated rates of cognitive change. However, it is unlikely that a retest effect affected the separation of participants into trajectory groups, and it could not have influenced the estimated association of sociodemographic, clinical, and risk factors variables with change. Forth, immediate and delayed verbal memory was assessed using one indicator, the CERAD list. It would be of interest to include more assessment tools to evaluate cognitive domains to analyze if those differences could be due to task-specific effects. Notwithstanding, the CERAD list is a standard test which has been used in other of trajectories of verbal memory [11,12,13]. Lastly, the study of cognitive trajectories and the time-varying variables could have been analyzed more in-depth using linear mixed models or a growth mixture model (GMM) [30]. Nevertheless, since we aimed to identify groups of individuals according to their immediate and delayed verbal memory scores without taking into account covariates, and to allow comparability among previous studies on cognitive trajectories [11,12], we first considered the GMM approach. However, the GMM failed due to non-convergence issues. So, we considered the LCGA/GBTM, a constrained GMM which assumes that all individual growth trajectories within classes are homogeneous. This approach is useful to capture the heterogeneity in individual trajectories and to obtain meaningful latent groups of individuals following similar paths in the outcome of interest, in terms of descriptive analysis [48,49]. Whether the identified trajectories are mainly descriptive, the study of modifiable risk factors associated with those groups is essential to detect groups of individuals to create proper prevention and intervention programs to delay the onset or rate of cognitive decline [50].

The strengths of this work include its being based on a large nationally-representative sample of community-dwelling individuals. In addition, due to the longitudinal nature of the study, we analyzed the same cohort for 7 years, thereby allowing the study of verbal memory trajectories to take into account cohort effects, in contrast to the cross-sectional design, which overestimates the amount of decline [40]. Furthermore, we examined the relationships among sociodemographic data, clinical variables, health-related behaviors, and each verbal memory trajectory, in order to determine which variables might be related to different cognitive profiles in the two age-groups. Moreover, we performed the analyses for both middle-aged and older individuals, to look for differences between those age groups, because most of studies have focused on older individuals [11,40]. In a similar way, we drew a distinction between immediate and delayed verbal memory, since they reflect different cognitive domains which could decline in different ways [16,36]. To our knowledge, no previous study has differentiated between the two measures to study verbal memory trajectories. 

## 5. Conclusions

This study has several potentially important clinical, research, and policy implications. Overall, our results suggest that decline is not exclusive to older people, although it was more common in that age group since only declining trajectories were identified. In this respect, not great differences have been found in terms of rates of decline. However, we expect to find more pronounced differences as more assessments were included, since a longer follow-up would be the proper way to study changes in these cognition trajectories. Nonetheless, according to the systematic review of Karr et al. (2018), change points for cognitive abilities ranged from 3 to 7 years before mild cognitive impairment diagnosis. Thus the study of preclinical differences could be useful on the early detection of cognitive change [50].

In addition, we studied some associated sociodemographic, clinical, and health-related variables for each cognition trajectory. Education seems to be related to initial performance but its effect on the rate of decline needs to be studied in greater depth. Cardiovascular diseases, a greater number of CCs, and physical inactivity were strongly associated with lowered memory functioning in early ages. So, preventive and intervention programs based on the body of evidence on this topic should be designed to reduce cognitive decline and prevent dementia, as well as to promote healthier lifestyles, especially at earlier ages when health status is more reversible. 

## Figures and Tables

**Table 1:** Baseline characteristics of the sample by age category (*n* = 1089).

Characteristics	50–64 (*n* = 633)	65+ (*n* = 456)	*p*-Value ^1^
Age, mean [IQR]	56.62 [60.00, 53.00]	73.01 [77.00, 68.00]	<0.001
Women, *n* (%)	329 (52.0)	249 (54.6)	0.236
Marital status, *n* (%)			<0.001
Never married	65 (10.3)	29 (6.4)	
Married	453 (71.6)	268 (58.8)	
Divorced	68 (10.7)	15 (3.3)	
Widowed	47 (7.4)	144 (31.6)	
Level of education, *n* (%)			<0.001
Less than primary education	105 (16.6)	212 (46.5)	
Primary education	198 (31.3)	136 (28.8)	
Secondary education	225 (35.6)	76 (16.7)	
Tertiary education	105 (16.6)	32 (7.0)	
Ever worked, *n* (%)	587 (92.7)	365 (80.0)	<0.001
Household income quintiles, *n* (%)			<0.001
1st (lowest)	108 (17.1)	70 (15.4)	
2nd	111 (17.5)	111 (24.3)	
3rd	105 (16.6)	99 (21.7)	
4th	112 (17.7)	100 (21.9)	
5th (highest)	118 (18.6)	39 (8.6)	
Urban, *n* (%)	543 (85.8)	390 (85.5)	0.399
No. diseases, mean [IQR]	0.95 [1.00, 0.00]	1.52 [2.00, 1.00]	<0.001
Diseases, *n* (%)			
Diabetes	73 (11.5)	102 (22.4)	<0.001
Hypertension	217 (34.3)	224 (49.1)	<0.001
Asthma	38 (6.0)	47 (10.3)	0.002
COPD	35 (5.5)	57 (12.5)	<0.001
Arthritis	124 (19.6)	153 (33.4)	<0.001
Angina pectoris	27 (4.3)	41 (9.0)	<0.001
Stroke	18 (2.8)	27 (6.0)	<0.001
Depression	72 (11.4)	47 (10.3)	0.453
Tobacco, *n* (%)			<0.001
Never smoked	273 (43.1)	280 (61.4)	
Daily smoker	169 (26.7)	41 (9.0)	
Not daily smoker	14 (2.2)	6 (1.3)	
Not current smoker	177 (28.0)	129 (28.3)	
Alcohol, *n* (%)			<0.001
Lifetime abstainers	168 (26.5)	158 (34.7)	
Occasional drinkers	170 (26.9)	132 (29.0)	
Infrequent heavy drinker	11 (1.7)	2 (0.4)	
Frequent heavy drinker	284 (44.9)	164 (36.0)	
Physical activity level, *n* (%)			<0.001
Low	199 (31.4)	134 (29.4)	
Moderate	225 (35.6)	201 (44.1)	
High	209 (33.0)	121 (26.5)	
WHODAS, mean [IQR]	7.60 [8.33, 0.00]	15.02 [25.00, 0.00]	<0.001
WHOQOL-Age, mean [IQR]	73.68 [83.10, 66.67]	72.54 [82.42, 64.15]	<0.001
Immediate verbal memory, mean [IQR]	17.36 [21.00, 14.00]	13.41 [16.00, 10.00]	<0.001
Delayed verbal memory, mean [IQR]	5.35 [7.00, 4.00]	3.74 [5.00, 2.00]	<0.001

Note. Household income was divided into 5 quintiles (the first indicating the lowest income). Marital status ‘married’ category included ‘currently married or cohabiting’, and ‘divorced’ included ‘divorced or separated’. Abbreviations: IQR, interquartile range; COPD, chronic obstructive pulmonary disease. ^1^ Based on Mann–Whitney U tests for numerical variables and Chi-squared tests for categorical variables.

**Table 2:** Model comparison of immediate and delayed verbal memory according to adjusted Bayesian information criterion (aBIC) over individuals aged 50–64 and 65+.

Age Groups	No. of Latent Classes	Immediate Verbal Memory		Delayed Verbal Memory	
		aBIC	Entropy	aBIC	Entropy
50–64	2	11125.778	0.676	8037.546	0.726
	3	11008.528	0.706	7971.521	0.691
	**4**	**10999.151**	**0.688**	**7955.357**	**0.653**
	5	11002.767	0.681	7955.693	0.69
65+	2	7722.539	0.646	5555.695	0.692
	**3**	**7680.159**	**0.654**	**5541.606**	**0.547**
	4	7674.616	0.698	5541.611	0.642
	5	7673.520	0.712	5542.644	0.626

Note. Boldface indicates the final selected model. *aBIC* adjusted Bayesian information criterion.

**Table 3:** Comparison of sociodemographic and clinical characteristics between immediate verbal memory trajectories in the 50–64 subsample.

		*50–64*				
	“Very Low/Decline” (*n* = 31, 6.3%)	“Low/Stable” (*n* = 248, 38.2%)	“Medium/Slow Decline” (*n* = 281, 43.4%)	“High/Slow Decline” (*n* = 73, 12.2%)	*p*-Value ^1^	*Effect Size* ^2^
Age	58.41 [61.5, 55.5]	57.85 [61.0, 55.0]	55.75 [59.0, 52.0]	55.01 [57.0, 52.0]	<0.001	0.07
Women, *n* (%)	13 (41.9)	118 (47.6)	152 (54.1)	46 (63.0)	0.064	0.10
Marital status, *n* (%)					0.104	0.09
Never married	5 (16.1)	20 (8.1)	25 (9.0)	15 (20.6)		
Married	20 (64.5)	186 (75.0)	203 (72.2)	44 (60.3)		
Divorced	4 (13.0)	20 (8.1)	36 (12.8)	8 (11.0)		
Widowed	2 (6.5)	22 (8.9)	17 (6.1)	6 (8.2)		
Level of education, *n* (%)					<0.001	0.32
Less than primary education	15 (48.4)	67 (27.0)	22 (7.8)	1 (1.4)		
Primary education	14 (45.2)	104 (41.9)	73 (26.0)	7 (9.6)		
Secondary education	1 (3.2)	60 (24.2)	138 (49.1)	26 (35.6)		
Tertiary education	1 (3.2)	17 (6.9)	48 (17.1)	39 (53.4)		
Ever worked, *n* (%)	28 (90.3)	219 (88.3)	267 (95.0)	73 (100.0)	0.001	0.15
Household income quintiles, *n* (%)					<0.001	0.17
1st (lowest)	3 (13.6)	38 (17.4)	51 (20.6)	16 (24.6)		
2nd	6 (27.3)	55 (25.1)	36 (14.5)	14 (21.5)		
3rd	6 (27.3)	54 (24.7)	40 (16.1)	5 (7.7)		
4th	7 (31.8)	45 (20.6)	54 (21.8)	6 (9.2)		
5th (highest)	0 (0.0)	27 (12.3)	67 (27.0)	24 (36.9)		
Urban, *n* (%)	21 (67.7)	215 (86.7)	241 (85.8)	66 (90.4)	0.021	0.12
No. Diseases	1.52 [3.0, 1.5]	1.17 [3.0, 1.0]	0.76 [2.0, 1.0]	0.66 [2.0, 1.0]	<0.001	0.04
Diseases, *n* (%)						
Diabetes	7 (22.6)	41 (16.5)	18 (6.4)	7 (9.6)	<0.001	0.16
Hypertension	13 (43.3)	93 (38.8)	93 (33.7)	18 (25.0)	0.123	0.09
Asthma	2 (6.5)	19 (7.7)	12 (4.3)	5 (6.9)	0.422	0.06
COPD	3 (9.7)	20 (8.1)	10 (3.6)	2 (2.7)	0.064	0.10
Arthritis	9 (29.0)	64 (25.8)	44 (15.6)	7 (9.6)	0.001	0.15
Angina pectoris	2 (6.9)	12 (5.1)	10 (3.6)	3 (4.2)	0.783	0.04
Stroke	3 (9.7)	9 (3.6)	6 (2.14)	0 (0.0)	0.037	0.11
Depression	8 (25.8)	37 (14.9)	21 (7.5)	6 (8.2)	0.002	0.15
Tobacco, *n* (%)					0.495	0.06
Never smoked	17 (54.8)	114 (46.0)	114 (40.6)	28 (38.4)		
Daily smoker	7 (22.6)	62 (25.0)	80 (28.5)	20 (27.4)		
Not daily smoker	1 (3.2)	4 (1.6)	5 (1.8)	4 (5.5)		
Not current smoker	6 (19.4)	68 (27.4)	82 (29.2)	21 (28.8)		
Alcohol, *n* (%)					0.165	0.17
Lifetime abstainer	8 (25.8)	81 (32.7)	63 (22.4)	16 (21.9)		
Occasional drinker	9 (29.0)	69 (27.8)	77 (27.4)	15 (20.6)		
Infrequent heavy drinker	0 (0.0)	5 (2.0)	6 (2.1)	0 (0.0)		
Frequent heavy drinker	14 (45.2)	93 (37.5)	135 (48.0)	42 (57.5)		
Physical activity level, *n* (%)					0.001	0.13
Low	15 (48.4)	86 (34.7)	85 (30.3)	13 (17.8)		
Moderate	10 (32.3)	85 (34.3)	109 (38.8)	21 (28.8)		
High	6 (19.4)	77 (31.0)	87 (31.0)	39 (53.4)		
WHODAS	12.72 [18.0, 0.0]	9.84 [13.8, 0.0]	5.73 [5.5, 0.0]	5.02 [2.7, 0.0]	<0.001	0.04
WHOQOL-Age	63.34 [74.2, 50.7]	71.88 [80.0, 65.0]	75.00 [84.5, 67.7]	79.88 [90.2, 71.3]	<0.001	0.04
Immediate verbal memory	10.68 [13.0, 8.5]	14.09 [17.0, 12.0]	18.95 [21.0, 17.0]	25.18 [27.0, 23.0]	<0.001	0.54
Delayed verbal memory	2.94 [4.0, 1.5]	4.28 [5.0, 3.0]	5.91 [7.0, 5.0]	7.88 [9.0, 7.0]	<0.001	0.32

Note. Values for continuous variables are mean and interquartile range [IQR]. *WHODAS* World Health Organization Disability Assessment Schedule 2.0, *WHOQOL-Age* World Health Organization Quality of Life instrument. ^1^ Based on Kruskal–Wallis tests for numerical variables and Chi-squared tests for categorical variables. ^2^ Based on η^2^ measured for Kruskal–Wallis for numerical variables and Phi coefficient for Chi-squared tests for categorical variables

**Table 4:** Comparison of sociodemographic and clinical characteristics between immediate verbal memory trajectories in the 65+ subsample.

		*65+*			
	“Low/Decline” (*n* = 87, 20.4%)	“Medium/Slow Decline” (*n* = 287, 60.4%)	“High/Slow Decline” (*n* = 82, 19.2%)	*p*-Value ^1^	*Effect Size* ^2^
Age	76.10 [79.3, 73.0]	72.80 [77.0, 68.0]	70.68 [73.0, 67.0]	<0.001	0.08
Women, *n* (%)	54 (62.1)	154 (53.7)	41 (50.0)	0.251	0.08
Marital status, *n* (%)				0.202	0.11
Never married	6 (7.0)	17 (5.6)	7 (8.5)		
Married	53 (61.0)	165 (57.5)	50 (61.0)		
Divorced	1 (1.2)	8 (2.8)	6 (7.3)		
Widowed	27 (31.0)	98 (34.2)	19 (23.2)		
Level of education, *n* (%)				<0.001	0.33
Less than primary education	59 (67.8)	140 (48.8)	13 (15.9)		
Primary education	24 (27.6)	94 (32.2)	18 (22.0)		
Secondary education	4 (4.6)	37 (12.9)	35 (42.7)		
Tertiary education	0 (0.0)	16 (5.6)	16 (19.5)		
Ever worked, *n* (%)	61 (70.1)	228 (79.4)	76 (92.7)	0.001	0.17
Household income quintiles, *n* (%)				<0.001	0.22
1st (lowest)	7 (9.3)	47 (17.6)	16 (20.8)		
2nd	32 (42.7)	73 (27.3)	6 (7.8)		
3rd	20 (26.7)	65 (24.3)	14 (18.2)		
4th	10 (13.3)	65 (24.3)	25 (32.5)		
5th (highest)	6 (8.0)	17 (6.4)	16 (20.8)		
Urban, *n* (%)	67 (77.0)	250 (87.1)	73 (89.0)	0.039	0.12
No. Diseases	1.64 [3.0, 2.0]	1.53 [3.0, 2.0]	1.35 [3.0, 2.0]	0.430	0.14
Diseases, *n* (%)					
Diabetes	28 (32.2)	64 (22.3)	10 (12.2)	0.007	0.15
Hypertension	38 (45.8)	143 (51.1)	43 (53.1)	0.609	0.05
Asthma	7 (8.1)	34 (11.9)	6 (7.3)	0.366	0.07
COPD	11 (12.6)	35 (12.2)	11 (13.4)	0.956	0.01
Arthritis	39 (44.8)	92 (32.1)	22 (26.8)	0.031	0.12
Angina pectoris	10 (12.5)	24 (8.8)	7 (8.97)	0.605	0.05
Stroke	6 (6.9)	15 (5.2)	6 (7.3)	0.710	0.04
Depression	11 (12.6)	32 (11.1)	4 (4.9)	0.187	0.09
Tobacco, *n* (%)				0.013	0.13
Never smoked	64 (73.6)	176 (61.3)	40 (48.8)		
Daily smoker	3 (3.5)	31 (10.8)	7 (8.5)		
Not daily smoker	2 (2.3)	3 (1.1)	1 (1.2)		
Not current smoker	18 (20.7)	77 (26.8)	34 (41.5)		
Alcohol, *n* (%)				0.023	0.14
Lifetime abstainer	43 (49.4)	96 (33.5)	19 (23.2)		
Occasional drinker	22 (25.3)	87 (30.3)	23 (28.1)		
Infrequent heavy drinker	0 (0.0)	2 (0.7)	0 (0.0)		
Frequent heavy drinker	22 (25.3)	102 (35.5)	40 (48.8)		
Physical activity level, *n* (%)				0.004	0.13
Low	36 (41.5)	82 (28.6)	16 (19.5)		
Moderate	36 (41.4)	131 (45.6)	34 (41.5)		
High	15 (17.2)	74 (25.8)	32 (39.0)		
WHODAS	24.10 [39.5, 5.5]	14.44 [25.0, 0.0]	8.06 [11.1, 0.0]	<0.001	0.07
WHOQOL-Age	66.15 [75.0, 59.2]	73.01 [80.9, 65.3]	77.23 [86.9, 68.3]	<0.001	0.06
Immediate verbal memory	9.49 [12.0, 7.0]	13.02 [15.0, 11.0]	18.62 [21.0, 16.0]	<0.001	0.38
Delayed verbal memory	2.59 [4.0, 1.75]	3.54 [4.0, 2.0]	5.59 [7.0, 4.0]	<0.001	0.22

Note. Values for continuous variables are mean and interquartile range [IQR]. *WHODAS* World Health Organization Disability Assessment Schedule 2.0, *WHOQOL-Age* World Health Organization Quality of Life instrument. ^1^ Based on Kruskal–Wallis tests for numerical variables and Chi-squared tests for categorical variables. ^2^ Based on η^2^ measured for Kruskal–Wallis for numerical variables and Phi coefficient for Chi-squared tests for categorical variables.

**Table 5:** Comparison of sociodemographic and clinical characteristics between delayed verbal memory trajectories in the 50–64 subsample.

		*50–64*				
	“Very Low/Decline” (*n* = 68, 12.4%)	“Low/Stable” (*n* = 346, 51.4%)	“Medium/Accelerated Decline” (*n* = 150, 24.7%)	“High/Slow Increase” (*n* = 69, 11.4%)	*p*-Value ^1^	*Effect Size* ^2^
Age	58.94 [62.0, 56.0]	56.91 [60.0, 54.0]	55.64 [59.0, 52.0]	54.97 [58.0, 51.0]	<0.001	0.07
Women, *n* (%)	26 (38.2)	170 (49.1)	84 (56.0)	49 (71.0)	<0.001	0.17
Marital status, *n* (%)					0.858	0.06
Never married	7 (10.3)	32 (9.3)	17 (11.3)	9 (13.0)		
Married	49 (72.1)	249 (72.0)	107 (71.3)	48 (69.6)		
Divorced	7 (10.3)	36 (10.4)	19 (12.7)	6 (8.7)		
Widowed	5 (7.4)	29 (8.4)	7 (4.7)	6 (8.7)		
Level of education, *n* (%)					<0.001	0.25
Less than primary education	18 (26.5)	72 (20.8)	11 (7.3)	4 (5.8)		
Primary education	39 (57.4)	117 (33.8)	32 (21.3)	10 (14.5)		
Secondary education	9 (13.2)	121 (35.0)	71 (47.3)	24 (34.8)		
Tertiary education	2 (3.0)	36 (10.4)	36 (24.0)	31 (44.9)		
Ever worked, *n* (%)	60 (88.2)	318 (91.9)	141 (94.0)	68 (98.6)	0.101	0.10
Household income quintiles, *n* (%)					<0.001	0.15
1st (lowest)	9 (15.8)	54 (17.6)	33 (25.0)	12 (20.7)		
2nd	20 (35.1)	58 (18.9)	22 (16.7)	11 (19.0)		
3rd	16 (28.1)	66 (21.5)	16 (12.1)	7 (12.1)		
4th	6 (10.5)	75 (24.4)	23 (17.4)	8 (13.8)		
5th (highest)	6 (10.5)	54 (17.6)	38 (28.8)	20 (34.5)		
Urban, *n* (%)	53 (78.0)	290 (84.0)	139 (92.7)	61 (88.4)	0.013	0.13
No. Diseases	2.29 [3.0, 1.0]	1.99 [2.0, 1.0]	1.84 [2.0, 1.0]	1.61 [2.0, 1.0]	0.135	0.05
Diseases, *n* (%)						
Diabetes	14 (20.6)	41 (11.9)	14 (9.3)	4 (5.8)	0.037	0.12
Hypertension	30 (45.5)	123 (36.3)	47 (32.2)	17 (25.4)	0.084	0.10
Asthma	3 (4.4)	26 (7.5)	4 (2.7)	5 (7.3)	0.182	0.09
COPD	4 (5.9)	24 (6.9)	6 (4.0)	1 (1.5)	0.240	0.08
Arthritis	18 (26.5)	68 (19.7)	29 (19.3)	9 (13.0)	0.269	0.08
Angina pectoris	3 (4.6)	15 (4.5)	7 (4.8)	2 (2.9)	0.937	0.03
Stroke	6 (8.8)	9 (2.6)	3 (2.0)	0 (0.0)	0.010	0.13
Depression	10 (14.7)	41 (11.9)	17 (11.3)	4 (5.8)	0.398	0.07
Tobacco, *n* (%)					0.060	0.09
Never smoked	26 (38.2)	163 (47.1)	60 (40.0)	24 (34.8)		
Daily smoker	18 (26.5)	92 (26.6)	38 (25.3)	21 (30.4)		
Not daily smoker	1(1.5)	6 (1.7)	2 (1.3)	5 (7.3)		
Not current smoker	23 (33.8)	85 (24.6)	50 (33.3)	19 (27.5)		
Alcohol, *n* (%)					0.109	0.09
Lifetime abstainer	18 (26.5)	92 (26.6)	39 (26.0)	19 (27.5)		
Occasional drinker	14 (20.6)	105 (30.4)	37 (24.7)	14 (20.3)		
Infrequent heavy drinker	4 (5.9)	3 (0.9)	4 (2.7)	0 (0.0)		
Frequent heavy drinker	32 (47.1)	146 (42.2)	70 (46.7)	36 (52.2)		
Physical activity level, *n* (%)					<0.001	0.14
Low	29 (42.7)	117 (33.8)	42 (28.0)	11 (15.9)		
Moderate	21 (30.9)	128 (37.0)	56 (37.3)	20 (29.0)		
High	18 (26.5)	101 (29.2)	52 (34.7)	38 (55.1)		
WHODAS	10.91 [16.7, 0.0]	7.99 [11.1, 0.0]	6.82 [8.3, 0.0]	4.11 [2.8, 0.0]	0.005	0.02
WHOQOL-Age	70.01 [77.7, 65.8]	72.91 [82.9, 66.2]	74.58 [82.5, 67.0]	79.21 [88.6, 70.6]	0.002	0.02
Immediate verbal memory	12.41 [15.0, 10.0]	15.84 [19.0, 13.0]	20.45 [23.0, 18.0]	23.16 [27.0, 21.0]	<0.001	0.37
Delayed verbal memory	2.81 [4.0, 2.0]	4.45 [5.0, 4.0]	7.23 [8.0, 6.0]	8.28 [10.0, 7.0]	<0.001	0.60

Note. Values for continuous variables are mean and interquartile range [IQR]. *WHODAS* World Health Organization Disability Assessment Schedule 2.0, *WHOQOL-Age* World Health Organization Quality of Life instrument. ^1^ Based on Kruskal–Wallis tests for numerical variables and Chi-squared tests for categorical variables. ^2^ Based on η^2^ measured for Kruskal–Wallis for numerical variables and Phi coefficient for Chi-squared tests for categorical variables.

**Table 6:** Comparison of sociodemographic and clinical characteristics between delayed verbal memory trajectories in the 65+ subsample.

		*65+*			
	“Low/Slow Decline” (*n* = 138, 34.4%)	“Medium/Decline” (*n* = 263, 52.7%)	“High/Slow Decline” (*n* = 55, 12.9%)	*p*-Value ^1^	*Effect Size* ^2^
Age	75.46 [79.3, 70.0]	72.23 [76.0, 68.0]	70.93 [73.0, 67.3]	<0.001	0.08
Women, *n* (%)	74 (53.6)	141 (53.6)	34 (61.8)	0.518	0.05
Marital status, *n* (%)				0.013	0.15
Never married	8 (5.8)	16 (6.1)	5 (9.1)		
Married	71 (51.5)	167 (63.5)	30 (54.6)		
Divorced	3 (2.2)	8 (3.0)	4 (7.3)		
Widowed	56 (40.6)	72 (27.4)	16 (29.1)		
Level of education, *n* (%)				<0.001	0.19
Less than primary education	80 (58.0)	116 (44.1)	16 (29.1)		
Primary education	43 (31.2)	80 (30.4)	13 (23.6)		
Secondary education	9 (6.5)	47 (17.9)	20 (36.4)		
Tertiary education	6 (4.4)	20 (7.6)	6 (11.0)		
Ever worked, *n* (%)	101 (73.2)	217 (82.5)	47 (85.5)	0.048	0.12
Household income quintiles, *n* (%)				0.345	0.10
1st (lowest)	17 (13.8)	44 (18.0)	9 (17.3)		
2nd	42 (34.2)	61 (25.0)	8 (15.4)		
3rd	28 (22.8)	59 (24.2)	12 (23.1)		
4th	25 (20.3)	58 (23.8)	17 (32.7)		
5th (highest)	11 (9.0)	22 (9.0)	6 (11.5)		
Urban, *n* (%)	108 (78.3)	232 (88.2)	50 (90.9)	0.012	0.14
No. Diseases	2.63 [3.0, 2.0]	2.49 [3.0, 2.0]	2.35 [3.0, 1.0]	0.395	0.13
Diseases, *n* (%)					
Diabetes	37 (26.8)	57 (21.7)	8 (14.6)	0.166	0.09
Hypertension	67 (50.0)	131 (51.0)	26 (49.1)	0.960	0.01
Asthma	16 (11.6)	27 (10.3)	4 (7.3)	0.671	0.04
COPD	23 (16.7)	26 (9.9)	8 (14.6)	0.132	0.09
Arthritis	48 (34.8)	90 (34.2)	15 (27.3)	0.571	0.05
Angina pectoris	11 (8.7)	24 (9.6)	6 (11.5)	0.837	0.03
Stroke	11 (8.0)	13 (5.0)	3 (5.5)	0.469	0.06
Depression	19 (13.8)	25 (9.5)	3 (5.5)	0.185	0.09
Tobacco, *n* (%)				0.138	0.10
Never smoked	89 (64.5)	159 (60.5)	32 (58.2)		
Daily smoker	8 (5.8)	31 (11.8)	2 (3.6)		
Not daily smoker	1 (0.7)	3 (1.1)	2 (3.6)		
Not current smoker	40 (29.0)	70 (26.6)	19 (34.6)		
Alcohol, *n* (%)				0.390	0.09
Lifetime abstainer	39 (40.6)	103 (31.2)	22 (36.4)		
Occasional drinker	43 (31.2)	76 (29.0)	13 (23.6)		
Infrequent heavy drinker	0 (0.0)	2 (0.8)	0 (0.0)		
Frequent heavy drinker	39 (28.3)	103 (39.2)	22 (40.0)		
Physical activity level, *n* (%)				0.003	0.13
Low	48 (34.8)	75 (28.5)	11 (20.0)		
Moderate	62 (44.9)	121 (46.0)	18 (32.7)		
High	28 (20.3)	67 (25.5)	26 (47.3)		
WHODAS	19.57 [33.3, 2.7]	13.66 [19.4, 0.0]	10.75 [15.9, 0.0]	<0.001	0.04
WHOQOL-Age	68.58 [78.9, 60.6]	73.61 [82.6, 65.4]	76.79 [86.4, 65.6]	<0.001	0.03
Immediate verbal memory	10.64 [13.0, 8.0]	13.91 [17.0, 11.0]	17.57 [21.0, 15.0]	<0.001	0.21
Delayed verbal memory	2.20 [3.0, 1.0]	3.97 [5.0, 3.0]	6.31 [7.8, 5.0]	<0.001	0.39

Note. Values for continuous variables are mean and interquartile range [IQR]. *WHODAS* World Health Organization Disability Assessment Schedule 2.0, *WHOQOL-Age* World Health Organization Quality of Life instrument. ^1^ Based on Kruskal–Wallis tests for numerical variables and Chi-squared tests for categorical variables. ^2^ Based on η^2^ measured for Kruskal–Wallis for numerical variables and Phi coefficient for Chi-squared tests for categorical variables.
